# Modulation of the Antitumor Response to Metformin, Caffeine, and Sodium Dichloroacetate by the Hypoxic Microenvironment in Lung Cancer Cells

**DOI:** 10.3390/ijms26115014

**Published:** 2025-05-23

**Authors:** Misael Osmar Garcia-Martin, Manuel Castillejos-Lopez, Heriberto Prado-Garcia, Susana Romero-Garcia, Juan Carlos Huerta-Cruz, José Alberto Choreño-Parra, Georgina Gonzalez-Avila, Luz A. Colín-Godínez, Daniel Paz-Gomez, Ángeles Carlos-Reyes, Victor Ruiz, Yair Romero, Edgar Flores-Soto, Juan Rodríguez-Silverio, Roberto Lara-Lemus, Rafael Velázquez-Cruz, Citlaltepetl Salinas-Lara, Luz María Torres-Espíndola, Arnoldo Aquino Gálvez

**Affiliations:** 1 Unidad de Epidemiologia Hospitalaria e Infectología, Instituto Nacional de Enfermedades Respiratorias Ismael Cosío Villegas (INER), Mexico City 14080, Mexico; docgamm@hotmail.com (M.O.G.-M.); mcastillejos@gmail.com (M.C.-L.); 2 Laboratorio de Onco-Inmunobiología, Departamento de Enfermedades Crónico-Degenerativas, Instituto Nacional de Enfermedades Respiratorias Ismael Cosío Villegas (INER), Mexico City 14080, Mexico; hpradog@yahoo.com (H.P.-G.); reyes_cardoso@yahoo.com (Á.C.-R.); 3 Laboratory of Immune System Biology, National Institute of Allergy and Infectious Diseases, National Institutes of Health, Bethesda, MD 20892, USA; 4 Unidad de Investigación en Farmacología, Instituto Nacional de Enfermedades Respiratorias Ismael Cosío Villegas (INER), Mexico City 14080, Mexico; hcjcarlosphd@hotmail.com (J.C.H.-C.); jrsilverio61@yahoo.com.mx (J.R.-S.); 5 Departamento de Enseñanza, Formación de Posgrado, Instituto Nacional de Enfermedades Respiratorias Ismael Cosío Villegas (INER), Calzada de Tlalpan 4502, Mexico City 14080, Mexico; choreprr@gmail.com; 6 Departamento de Enfermedades Crónico-Degenerativas, Instituto Nacional de Enfermedades Respiratorias Ismael Cosío Villegas (INER), Mexico City 14080, Mexico; ggonzalezavila@yahoo.com; 7 Departamento de Fibrosis Pulmonar, Laboratorio de Biología Molecular, Instituto Nacional de Enfermedades Respiratorias Ismael Cosío Villegas (INER), Mexico City 14080, Mexico; adriscg11@gmail.com (L.A.C.-G.); vicoruz@yahoo.com.mx (V.R.); 8 Laboratorio de Investigación en Enfermedades Reumáticas, Instituto Nacional de Enfermedades Respiratorias Ismael Cosío Villegas (INER), Mexico City 14080, Mexico; danielpazgmz@gmail.com; 9 Escuela Superior de Medicina, Sección de Estudios de Posgrado e Investigación, Instituto Politécnico Nacional (INP), Mexico City 11340, Mexico; 10 Facultad de Ciencias, Universidad Nacional Autónoma de México (UNAM), Mexico City 04510, Mexico; yair@ciencias.unam.mx; 11 Departamento de Farmacología, Facultad de Medicina, Universidad Nacional Autónoma de México (UNAM), Mexico City 04510, Mexico; edgarfloressoto@yahoo.com.mx; 12 Departamento de Biomedicina Molecular e Investigación Traslacional, Instituto Nacional de Enfermedades Respiratorias Ismael Cosío Villegas (INER), Mexico City 14080, Mexico; antonio.lara@iner.gob.mx; 13 Laboratorio de Genómica del Metabolismo Óseo, Instituto Nacional de Medicina Genómica (INMEGEN), Mexico City 14610, Mexico; rvelazquez@inmegen.gob.mx; 14 Laboratorio de Patología, Instituto Nacional de Neurología y Neurocirugía “Manuel Velazco Suarez” (INNNMVS), Mexico City 14269, Mexico; citlalsalinas69@gmail.com; 15 Laboratorio de Farmacología, Instituto Nacional de Pediatría (INP), Mexico City 04530, Mexico; 16 Facultad de Medicina, Departamento de Bioquímica, Universidad Nacional Autónoma de México (UNAM), Mexico City 04510, Mexico

**Keywords:** lung cancer, HCC827, caffeine, DCA, metformin, hypoxia, isobolographic analysis

## Abstract

Metformin, caffeine, and dichloroacetate (DCA) have shown antitumor effects. The hypoxic tumor microenvironment can modulate drug response. We aimed to analyze the interaction of metformin with caffeine or DCA in lung cancer cells (HCC827) under normoxia and hypoxia conditions. Cell viability was evaluated using the crystal violet assay after individual and combined drug treatment under normoxia (21% O_2_) and hypoxia (1% O_2_) conditions. Combination effects were analyzed using isobolographic analysis. The results show that under normoxia conditions, the combination of metformin with DCA (γ = 0.98 ± 0.35, *p* > 0.05) or caffeine (γ = 0.90 ± 0.34, *p* > 0.05) revealed additivity. However, in hypoxia, both combinations exhibited significant antagonism, with γ values appearing greater than one for metformin + DCA (γ = 4.20 ± 1.44, *p* < 0.05) and metformin + caffeine (γ = 2.88 ± 0.90, *p* < 0.05). Hypoxia significantly alters the pharmacological interaction of metformin with caffeine or DCA, which could limit their combined therapeutic potential in hypoxic tumors despite metformin’s activity in this environment. The importance of considering tumor oxygenation status in the design of combined therapies for lung cancer is emphasized.

## 1. Introduction

Lung adenocarcinoma, the most common histological subtype of lung cancer, has a poor prognosis in early clinical stages, with an overall 5-year survival rate ranging from 12% to 17% [1,2,3]. However, in Mexico, most patients are diagnosed in stages III–IV [4], which leads to a low 5-year survival rate, exacerbated by restricted access to timely, targeted therapies [5]. This situation highlights the urgent need to develop new and more accessible therapies that can be integrated into existing treatment algorithms. In searching for new therapies, drugs such as metformin, caffeine, and DCA have shown antitumor activity. Metformin, a widely used antidiabetic drug, acts through the activation of Adenosine Monophosphate-activated Protein Kinase (AMPK), inhibiting the Protein Kinase B/Mammalian Target of Rapamycin (AKT/mTOR) pathway and resulting in apoptosis and decreased cell proliferation [6,7]. Furthermore, it reduces c-MYC expression [8,9,10] and suppresses Hypoxia-Inducible Factor 1-alpha (HIF-1α) activation under hypoxic conditions [11]. Caffeine is a nervous system stimulant with an antitumor effect by inducing p53-dependent apoptosis via the inhibition of the Phosphoinositide 3-Kinase (PI3K) pathway [12,13,14] and reducing proliferation, invasion, and cell migration [15,16]. Finally, the pyruvate dehydrogenase kinase inhibitor DCA has shown antitumor potential by modifying the Warburg effect, reducing lactate and glucose consumption, and slowing cell growth in non-small cell lung cancer (NSCLC) [17]. The selection of metformin, caffeine, and DCA was based on their potential to regulate cell metabolism and, particularly, to influence mitochondrial respiration. Preclinical research suggests the potential effects of these drugs in lung cancer therapy (Table 1); however, evidence on the effectiveness of their combinations, especially under hypoxic conditions, remains limited. Hypoxia, common in the tumor microenvironment, has been strongly associated with resistance to conventional and biological chemotherapy by decreasing healing and survival rates [18,19].

To address this knowledge gap, in the present study, we performed an isobolographic analysis to test the pharmacological interaction of metformin with either caffeine or DCA under normoxia and hypoxia conditions in the lung adenocarcinoma cell line HCC827 (CRL-2868), which has an Epidermal Growth Factor Receptor (EGFR) mutation and sensitivity to Tyrosine Kinase Inhibitors (TKIs), In addition, the lower oxygen consumption of this cell line facilitated the induction and controlled maintenance of hypoxia, allowing for a more accurate simulation of the tumor microenvironment. Isobolographic analysis allows for the evaluation of the pharmacological interaction of two drugs using a two-dimensional (2D) graph, which includes isoeffect lines and a diagonal line representing the theoretical sum of the pharmacological effect [20]. By generating concentration–response curves for each drug and their combinations, it is possible to determine whether the interaction is additive, synergistic, or antagonistic. Isobolographic analysis is crucial for optimizing combination therapies by maximizing efficacy and minimizing toxicity [21]. Therefore, this study sought to evaluate the interaction of metformin, caffeine, and DCA in lung adenocarcinoma cells under normoxic and hypoxic conditions, using isobolographic analysis to provide insight into the role of hypoxia in combination therapies and allow hypoxia to be considered in future combinations.

**Table 1:** Antitumor effects of metformin, caffeine, and DCA, alone or its combination, under normoxic and hypoxic conditions. The main mechanisms of action and references are indicated.

Drug	Condition	Antitumor Effect	Mechanism	References
Metformin	Normoxia	Induces apoptosis Arrests the cell cycle Inhibits DNA repair Blocks mTOR/Akt	AMPK activation mTOR/Akt inhibition c-MYC reduction	[6,7,8,9,10,22,23,24,25]
Metformin	Hypoxia	Inhibits HIF-1α activation	Suppression of HIF-1α VEGF and MMP-2	[11,26]
Caffeine	Normoxia/ Hypoxia	Induces apoptosis Inhibits metastasis Reduces VEGF Increases cisplatin Inhibits integrin	Induction of p53 Inhibition of PI3K Adenosine receptor blockade	[12,13,14,15,16,27,28,29,30]
DCA	Normoxia/ Hypoxia	Modifies the Warburg effect Reduces lactate/glucose consumption Slows cell growth Synergizes with chemotherapy	Modification of tumor metabolism	[17,31,32,33,34,35]
Combinations				
Metformin + Caffeine	In vivo	Inhibit fibrosarcoma	Decreased Ki-67	[36]
Metformin + DCA	Normoxia	Additive effect Suppresses expansion Increases apoptosis Inhibits mTOR1 and MCL-1	Inhibición de mTOR1 y MCL-1	[33,37]
Metformin + DCA	Normoxia	Increases cell death	In the absence of HIF-1α	[38]

Abbreviations: Adenosine Monophosphate-activated Protein Kinase (AMPK), oncogenic transcription factor (c-MYC), Hypoxia-Inducible Factor 1-alpha (HIF-1α), Myeloid Cell Leukemia Sequence 1 (MCL-1), Matrix Metalloproteinase-2 (MMP-2), Phosphoinositide 3-Kinase (PI3K), and Vascular Endothelial Growth Factor (VEGF).

## 2. Results

### 2.1. Oxygen Consumption

**Figure 1:**
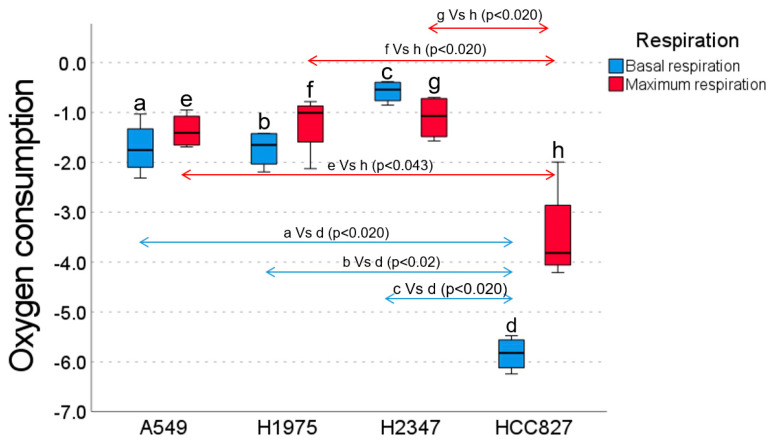
Basal (blue bars) and maximum (red bars) oxygen consumption rates (OCRs) of lung cancer cell lines: A549, H1975, H2347, and HCC827. The HCC827 cell line exhibited significantly lower basal OCRs compared to A549 (*p* < 0.020), H1975 (*p* < 0.020), and H2347 (*p* < 0.020). Conversely, H2347 displayed the highest basal OCR, which was significantly greater than A549 (*p* < 0.020) and H1975 (*p* < 0.020). Regarding the maximum OCR, HCC827 showed significantly lower rates compared to A549 (*p* < 0.020), H1975 (*p* < 0.043), and H2347 (*p* < 0.020).

The lowest oxygen consumption was observed in the HCC827 cell line, with a median of −5.83, with statistically significant differences compared to the other cell lines (Figure 1). The highest oxygen consumption was shown in the H2347 cell line, with a median of −0.54 (*p* < 0.05 compared to the other cell lines). The two remaining cell lines (A549 and H1975) with similar medians (−1.75 and −1.65, respectively, *p* > 0.05) showed intermediate oxygen consumption. Regarding maximum oxygen consumption (via glutamate-malate ADP binding), the HCC827 cell line had the lowest oxygen consumption, with a median of −3.81 and statistically significant differences (Figure 1). The rest of the cell lines (A549, H1975, H2347) maintained consumption with similar medians (−1.40, −1.00, −1.07, respectively, *p* > 0.05).

### 2.2. Pharmacological Effect of Metformin

**Figure 2:**
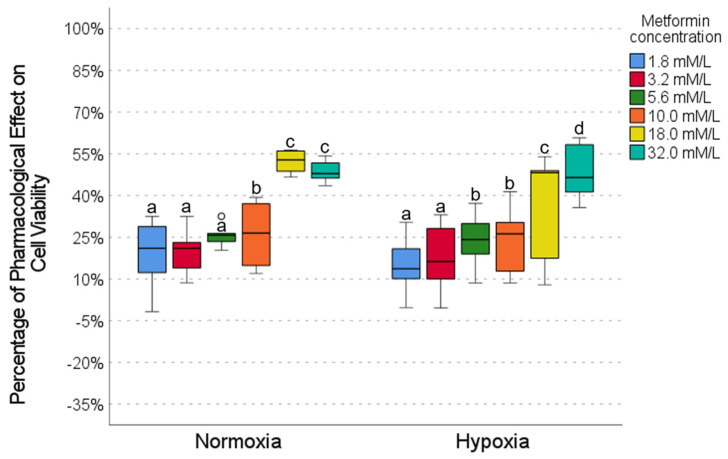
The effect of metformin on cell viability under normoxic and hypoxic conditions. Intragroup comparisons (between concentrations under the same oxygen conditions) were analyzed using the Wilcoxon test; statistical significance is represented by different letters above the bars (*p* < 0.05). Intergroup comparisons (the same concentration under normoxic vs. hypoxic conditions) were evaluated using the Mann–Whitney U test. The circles in the graph represent outliers identified during the analysis. Exact *p*-values for each statistical comparison are provided in the Supplementary Materials (Tables S1–S3). The Mann–Whitney U test revealed no statistically significant differences in cell viability between the same metformin concentrations when comparing normoxic and hypoxic conditions.

The effect of metformin on cell viability was evaluated at different concentrations under normoxic and hypoxic conditions (Figure 2). Under normoxia conditions, low metformin concentrations (1.8, 3.2, and 5.6 mmol) showed limited pharmacological activity (20–26%), with no significant differences between them, as shown in the *p*-value tables (Figure 2). In contrast, at high concentrations (18.0 and 32.0 mmol), metformin showed the highest activity (48–52%), which was significantly higher than at low concentrations (Figure 2). In hypoxia, low concentrations (1.8 and 3.2 mmol) also showed low activity (12–14%); intermediate concentrations (5.6 and 10.0 mmol) showed moderate activity (25–27%); and the 32.0 mmol concentration showed the highest activity (48%) (Figure 2). When comparing the effects of the same concentrations between normoxia and hypoxia, no significant differences were observed (Figure 2), indicating that the oxygenation condition did not substantially alter the response to metformin, so the effect was preserved regardless of hypoxia.

### 2.3. Pharmacological Effect of Caffeine

**Figure 3:**
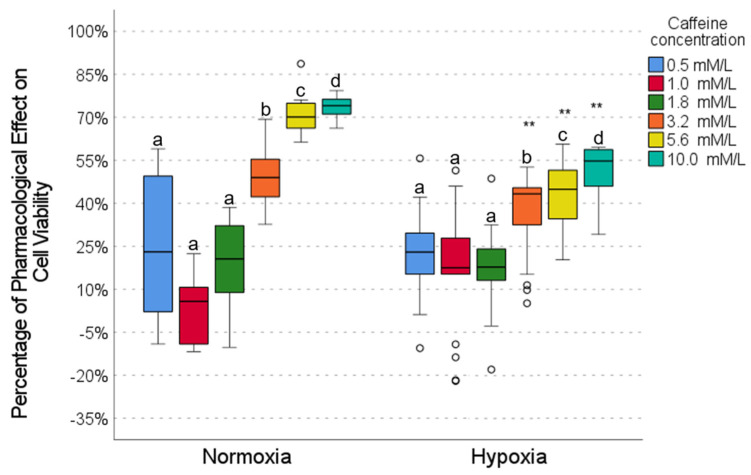
The effect of caffeine on cell viability under normoxic and hypoxic conditions. Intragroup comparisons (between concentrations under the same oxygen conditions) were analyzed using the Wilcoxon test; statistical significance is represented by different letters above the bars (*p* < 0.05). Intergroup comparisons (same concentration under normoxic vs. hypoxic conditions) were evaluated using the Mann–Whitney U test (** *p* < 0.01). The circles in the graph represent the outliers identified during the analysis. The exact *p*-values for each statistical comparison are provided in the Supplementary Materials (Tables S4–S6).

The effect of caffeine varied significantly depending on the concentration and oxygen conditions. In normoxia conditions, low concentrations (0.5, 1.0, and 1.8 mmol/L) showed minimal pharmacological activity (∼8–24%), with no significant differences between them (Figure 3). In contrast, high concentrations (5.6 and 10.0 mmol/L) demonstrated the most significant activity (∼72–74%), which was similar between both concentrations but significantly different from the low concentrations. In hypoxia, low concentrations also showed low activity (∼18–24%), with no differences between them. At high concentrations (5.6 and 10.0 mmol/L), caffeine maintained moderate activity (∼45–55%) but showed significantly higher levels of activity than the low concentrations (Figure 3). When comparing normoxia with hypoxia conditions, low concentrations showed no significant changes (Figure 3), while high concentrations showed a reduced activity in hypoxia (50–75% in normoxia vs. 40–55% in hypoxia). These results suggest that caffeine exerts a concentration-dependent pharmacological effect, attenuated under hypoxic conditions.

### 2.4. Pharmacological Effect of DCA

**Figure 4:**
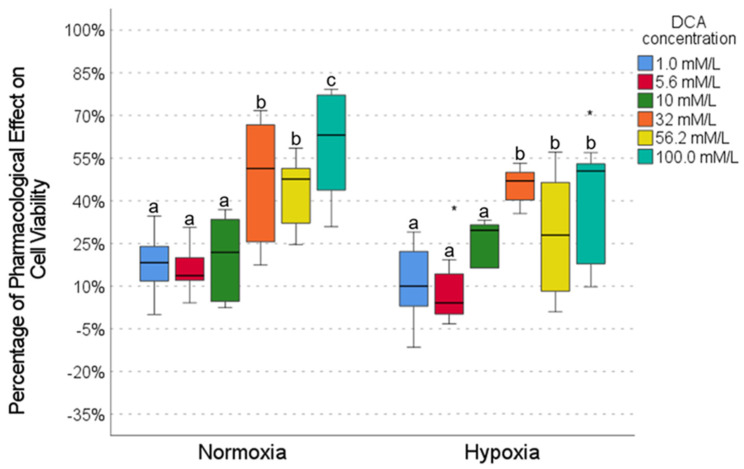
The effect of DCA on cell viability under normoxic and hypoxic conditions. Intragroup comparisons (between concentrations under the same oxygen conditions) were analyzed using the Wilcoxon test; statistical significance is represented by different letters above the bars (*p* < 0.05). Intergroup comparisons (same concentration under normoxic vs. hypoxic conditions) were evaluated using the Mann–Whitney U test (* *p* < 0.05). The circles in the graph represent outliers identified during the analysis. Exact *p*-values for each statistical comparison are provided in the Supplementary Materials (Tables S7–S9).

DCA exhibited a concentration-dependent pharmacological effect in the HCC827 cell line, with low activity (10–20%) at low concentrations (1.0, 5.6, and 10.0 mmol/L) and high activity at high concentrations (∼60% at 100.0 mmol), both in normoxia and hypoxia conditions (Figure 4); however, a significant decrease in the effect of DCA in hypoxia (5–20%) was observed for intermediate and high concentrations (5.6, 56.2, and 100.0 mmol, Figure 4), suggesting that the efficacy of DCA is attenuated by hypoxia.

### 2.5. Drug Combinations

To evaluate the potentiation or additive effects of the three drugs, an experimental approach focused on two drug combinations was chosen, prioritizing metformin due to its stability in hypoxia and clinical relevance, combining it with either DCA or caffeine. This strategy is based on experimental data suggesting potential synergies in the context of cellular metabolism and response to hypoxia and the widespread use and study of metformin and caffeine, thus seeking to maximize the efficiency of obtaining relevant information on drug interactions with the available experimental resources.

To determine the concentrations of the drugs in combination, individual concentration–response curves were generated for each drug under normoxic and hypoxic conditions. From these curves, the theoretically effective concentration of 50% (EC50) was calculated for each compound using the Hill equation and considering the maximum effect on the system. This approach allowed for the selection of EC50 values that represented comparable basal activity between the drugs. The goal was to achieve a theoretical combined effect of 50%, which would allow for potential potentiation or synergy between the drugs to be evaluated. The four concentrations of the combination of metformin with DCA and metformin with caffeine used in HCC827 cells in normoxia and hypoxia conditions were, thus, calculated and are shown in Table 2.

### 2.6. Pharmacological Interaction of Metformin with Caffeine in Normoxia and Hypoxia Conditions

**Figure 5:**
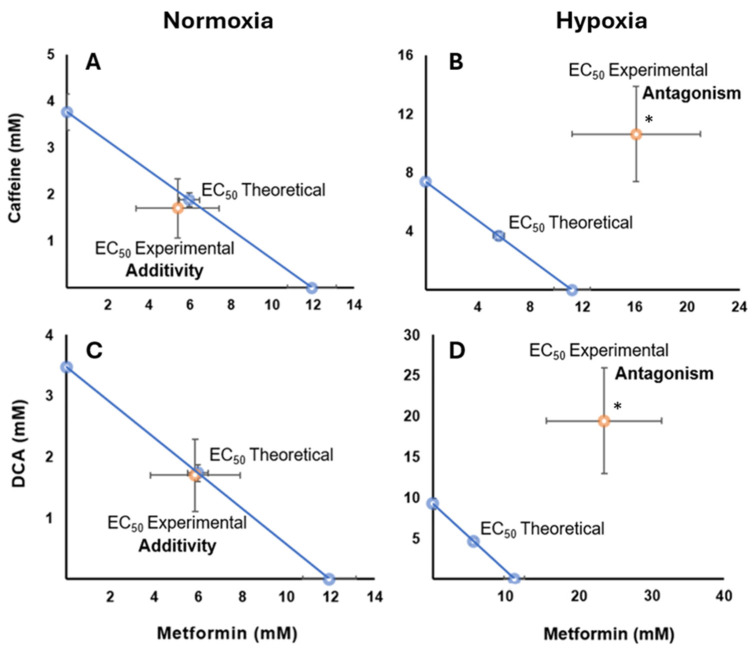
Isobolograms showing drug interactions in HCC827 cells under different oxygenation conditions. The diagonal blue line extending between the ordinate and abscissa axes connects the desired and effective concentration (EC50) of metformin with caffeine or DCA, representing the theoretical sum of the individual drug effects (the additivity line). (**A**) An additive effect is observed for the combination of metformin and caffeine in normoxia conditions, where the experimental points are located close to the additivity line. (**B**) The same combination in hypoxia shows an antagonistic effect, as evidenced by the deviation of the experimental point (orange) above the additivity line, indicating the need for higher concentrations to achieve the same effect. (**C**) The interaction between metformin and DCA in normoxia conditions presents an additive effect (*p* > 0.05), with the experimental points close to the theoretical line. (**D**) In contrast, the interaction between metformin and DCA in hypoxia exhibits a significant antagonistic effect (* *p* < 0.05), with the experimental points deviating away from the additivity line, suggesting a decreased combined effect compared to the sum of the individual effects.

Isobolographic analysis of the combination of metformin and caffeine in HCC827 cells cultured under normoxic conditions revealed an additive pharmacological effect, as indicated by an interaction index (γ) of 0.90 (*p* > 0.5). This is visually represented by the location of the experimental points near the theoretical additivity line (Figure 5A) without reaching statistical significance. However, under hypoxic conditions, a significant antagonistic effect was observed (γ = 2.88, *p* < 0.05) (Figure 5B), with the experimental points located clearly above the additivity line, demonstrating a statistically significant deviation from additivity. All values from the statistical analyses are provided in the Supplementary Materials (Tables S10 and S11).

### 2.7. Drug Interaction of Metformin with DCA Under Normoxia and Hypoxia Conditions

The combination of metformin with DCA showed an additive effect in normoxia, indicated by an interaction index (γ) of 0.98 (*p* > 0.5) (Figure 5C), with no significant differences from the theoretical additivity. However, in hypoxia conditions, a significant antagonistic effect was detected (γ = 4.20, *p* < 0.05) (Figure 5D), with a statistically significant deviation from the additivity line, as evidenced by the interaction index, which was significantly greater than one. All values from the statistical analyses are provided in the Supplementary Materials (Tables S12 and S13).

## 3. Discussion

The antitumor effects of metformin, caffeine, and DCA have been reported in several studies (Table 1). Our group is interested in identifying molecules capable of reversing the characteristic tumor-like phenotype of cancer cells, specifically their ability to maintain cell viability even under stress conditions such as hypoxia. In this context, it is relevant to investigate whether the effect of these compounds in normoxia conditions is maintained under hypoxic conditions and if their individual effects can be additive or synergistic under the two oxygenation conditions. Cell viability, measured for the crystal violet assay, is considered a primary indicator of the tumor phenotype that we sought to modulate in this study.

Like other researchers, we postulate that any molecule that can reverse glycolytic metabolism in tumors represents a potential drug or adjuvant in cancer treatment. Our main hypothesis was that the combination of metformin with caffeine or DCA would show a synergistic effect in inhibiting the cell viability of the HCC827 line and that this effect could be altered by hypoxic conditions.

Our results show that caffeine, metformin, and DCA exert concentration-dependent pharmacological effects on cell viability in the HCC827 cell line, which are more evident in normoxia than hypoxia conditions (Figure 2, Figure 3 and Figure 4). In this work, we highlight the importance of considering the tumor microenvironment by designing combination therapies for cancer. We also confirm that hypoxia alters drug response, affecting metabolic pathways modulated by metformin, caffeine, and DCA. It is important to highlight that metformin maintains its effect on cell viability even in a hypoxic environment (Figure 2).

Our experimental results, presented in the form of isobolographic analyses, reveal that metformin interacts differently with caffeine and DCA depending on the oxygenation conditions. Under normoxia conditions, the combination of metformin with either caffeine or DCA showed an additive effect (Figure 5A,C). This is consistent with previous evidence describing the antitumor effects of metformin, including the inhibition of cell proliferation and the induction of apoptosis, which are mechanisms that could be enhanced in the presence of caffeine or DCA [11,26,33,36,37,38]. Studies in other lung cancer cell lines have also reported additive effects of metformin with DCA under normoxic conditions, suggesting mechanisms related to the inhibition of mTOR1 and MCL-1 [33,37].

On the other hand, under hypoxic conditions, both combinations, i.e., metformin with caffeine and metformin with DCA, showed a significant antagonistic effect. This finding is particularly relevant given the crucial role of metformin in suppressing HIF-1α activation in hypoxia [11], suggesting that the hypoxic microenvironment could alter the signaling pathways and mechanisms of action of metformin when combined with caffeine or DCA. A possible mechanism for this antagonism could involve the differential modulation of AMPK activity by metformin in hypoxia conditions in the presence of caffeine or DCA. However, further studies are needed to confirm this hypothesis.

Metformin has consistently demonstrated antitumor effects in various cancer models, primarily through the induction of apoptosis and cell cycle arrest in lung cancer [22,23,24]. It inhibits DNA repair and blocks mTOR and Akt signaling pathways, resulting in apoptosis and decreased cell proliferation [6,7]. Furthermore, it reduces c-MYC expression by upregulating miR-33a, disrupting oncogenic metabolism, and stopping the cell cycle in G0/G1 [8,9,10]. A crucial aspect is its ability to suppress HIF-1α activation under hypoxic conditions [11], suggesting its potential as an adjuvant in chemotherapy, sensitizing head and neck cancer cells to gefitinib [39] and suppressing angiogenesis [40]. The relevance of hypoxia in response to metformin is highlighted by its ability to reduce the volume and weight of hepatocellular carcinoma tumors by decreasing the expression of HIF-1α, VEGF, and MMOLP-2 [26]. As we can see in our normoxia data, there is an additive effect of metformin with DCA, which is similar to a study where they observed that metformin shows an additive effect with DCA by inhibiting mTOR1 and MCL-1 [33,37]. However, our finding of antagonism in hypoxia with DCA differs from some previous studies suggesting greater DCA activity in hypoxia [34,35], which might reflect the specific characteristics of the HCC827 cell line or the specific experimental conditions of our study.

Caffeine induces apoptosis through the PI3K pathway [12,13] and p53 [14]; reduces cell proliferation and cancer cell spreading [15,27]; induces cell cycle arrest in cancer stem cells [16]; reduces VEGF activity in colon cancer under hypoxia conditions [28,29]; and potentiates the action of cisplatin in lung cancer [30], with in vivo evidence of fibrosarcoma inhibition when combined with metformin [36].

Finally, DCA has shown antitumor potential in NSCLCs [17], in addition to showing synergy with chemotherapeutics in NSCLCs [31,32] and with metformin in ovarian cancer [33], with greater activity in hypoxia [34,35]. However, in this work, we show that in HCC827 cells, the drug interaction of metformin with DCA shows an additive effect in normoxia conditions but a significant antagonistic effect in hypoxia conditions, suggesting that the tumor microenvironment, especially oxygenation, modulates the efficacy of this combination, highlighting the need for further research to optimize its clinical use. The antagonistic effect observed in the combination of metformin with DCA under hypoxic conditions could be due to the interference of DCA with metformin’s ability to suppress HIF-1α activation [11]. Given that HIF-1α can regulate the expression of metabolic enzymes and drug resistance factors in hypoxia, the alteration of its modulation by metformin in the presence of DCA could destabilize the metabolic balance induced by metformin or even favor pathways that counteract its antitumor effect. Our study’s contribution lies in observing this specific antagonistic effect under hypoxic conditions in the HCC827 cell line, which contrasts with some previous findings and underscores the complexity of drug interactions in different tumor microenvironments.

In hypoxia conditions, the combination of metformin with DCA shows drug antagonism, possibly due to its dependence on HIF-1α [38]. Hypoxia, a chemoresistance factor, involves mechanisms such as HIF-1α [41], microRNA suppression [25], MDR-1 overexpression [42,43,44,45,46], lactic acidosis [47,48], NF-κB overexpression [49], changes in mitochondrial dynamics [50,51], and dysregulated glycolysis [52]. The interrelation of these mechanisms in hypoxia could explain the antagonistic effect of our pharmacological combinations in this condition. Although we did not directly assess lactate production or glucose consumption in this study, as this was not its primary objective, the literature suggests that hypoxia induces changes in these metabolic pathways. These changes could influence the cellular response to metformin, caffeine, and DCA, potentially contributing to the observed antagonism. Future research could explore these specific pathways using our model.

It would be appropriate to explore whether metformin, caffeine, and DCA could function independently as potential adjuvants in standard cancer treatments, exploring whether their individual and combined antitumor effects are maintained or altered under hypoxic conditions. Understanding these interactions at different oxygen levels could optimize combination therapies to overcome tumor resistance. The limitations of this work are that the HCC827 cell line was derived from a lung adenocarcinoma with EGFR mutation and high sensitivity to TKIs, which prevents us from generalizing the results to other cancer types and tumor microenvironments. However, the choice of this cell line was justified due to its lower oxygen consumption, and the handling of multiple cell lines would have complicated proper management, given that experiments were performed in triplicate and with three drugs. In the future, it will be appropriate to expand the research to other cell lines with different metabolic characteristics to evaluate the robustness of the findings and their clinical relevance. Translating in vitro findings to in vivo models presents significant challenges due to the complexity of the real tumor microenvironment, which includes interactions with the stroma, the immune system, and vasculature. Therefore, validating the antitumor effects observed in HCC827 cells in animal models with solid tumors that reproduce hypoxia conditions and tumor heterogeneity is crucial. Furthermore, it should be considered that the pharmacokinetics and toxicity of drugs in a systemic context are necessary before advancing to clinical trials. In future research, it would be interesting to explore the specific molecular mechanisms responsible for the observed antagonism in hypoxia, perhaps by analyzing key signaling pathways such as AMPK, PI3K/Akt/mTOR, and HIF-1α under different oxygenation conditions and drug combinations.

## 4. Materials and Methods

### 4.1. Cell Culture

The human lung cancer cell line A549 (CCL-185™) was derived from a lung carcinoma of a 58-year-old White male; H1975 (CRL-5908™); an epithelial morphology cell line was isolated from the lungs of a nonsmoking female with non-small cell lung cancer; H2347 (CRL-5942™), also exhibiting epithelial morphology, was isolated from the lung of a 54-year-old White female patient with stage 1 non-small cell lung cancer; and HCC827 (CRL-2868™), an epithelial cell obtained from a lung adenocarcinoma of a 39-year-old White female, was purchased from the American Type Culture Collection (ATCC, Manassas, VA, USA). Cells were cultured in Dulbecco’s Modified Eagle Medium (DMEM) with high glucose levels (4.5 g/L) supplemented with 100 U/mL penicillin, 100 μg/mL streptomycin, 2.5 mg/mL amphotericin B (Gibco, Waltham, MA, USA), and 10% fetal bovine serum (Gibco) in an atmosphere of 95% air and 5% CO_2_ at 37 °C with controlled humidity in an incubator, following ATCC recommendations.

### 4.2. Cell Line Selection

The metabolic phenotype of four lung adenocarcinoma cell lines was estimated by measuring oxygen consumption. The cell line with the lowest oxygen consumption was selected, assuming a predominantly glycolytic metabolism, to evaluate the effect of drug combinations on cell growth. After 72 h of incubation in normoxia, cells were harvested by trypsinization, washed with PBS, and resuspended in a respiration buffer (120 mmol KCl, 10 mmol MgCl_2_•6H_2_O, 1 mmol EDTA, 8.1 mmol KH_2_PO_4_•7H_2_O, and 1.46 mmol K_2_HPO_4_, pH 7.4 with 10 M KOH) at a concentration of 1 × 10^6^ cells/300 μL. Oxygen consumption was measured using a Clark electrode and the YSI 5300A system (Xylem Analytics, Yellow Springs, OH, USA) at 37 °C with constant stirring, calibrating the system according to the manufacturer. In total, 300 μL of the cell suspension was placed in the chamber with 700 μL of the oxygen-saturated respiration buffer at 37 °C, resulting in 1 × 10^6^ cells/mL. Oxygen concentration was recorded every second with Hterm v. 0.8.1, obtaining the basal respiration rate before the permeabilization of the cells with digitonin [53] (7.5 μg/mL, 5 min). Glutamate (10 mmol), malate (5 mmol), and ADP/MgCl_2_ (1 mmol) were added to evaluate the respiratory chain [10]. Maximum oxygen saturation was calibrated at 400 nM O_2_/mL. Oxygen consumption (nM O_2_/mL per 1 × 10^7^ cells) was graphed, and analyses were performed in triplicate. Therefore, based on the previous analysis, it was decided to use the cell line that respired the least, HCC827 (CRL-2868™), which was a lung adenocarcinoma cell line with adherent epithelial cell characteristics extracted from a 39-year-old Caucasian woman.

### 4.3. Drugs

Caffeine (Cat. C0750-100G), metformin hydrochloride (1,1-dimethylbiguanide) (Cat. D150959-5G), and DCA (Cat. 347795-10G) were purchased from Sigma-Aldrich Co. (St. Louis, MO, USA). Given the water solubility of these compounds, the DMEM culture medium was used as a solvent, eliminating the need for organic solvents. The concentrations of each drug for the concentration–response curves were determined from a thorough review of the scientific literature and were considered on a logarithmic scale. Specifically, the following concentrations were used: caffeine (0.5, 1, 1.8, 3.2, 5.6, 10 mmol), metformin (1.8, 3.2, 5.6, 10, 18, 32 mmol), and DCA (1, 5.6, 10, 32, 56.2, 100 mmol).

### 4.4. Hypoxia

To promote hypoxia, the cultures were placed in a modular chamber (Billups-Rothenberg Inc., San Diego, CA, USA) and placed in a culture incubator with a constant temperature of 37 °C for 72 h. The atmosphere within the chamber was controlled to maintain a humidified gas mixture composed of 1% O_2_ and 5% CO_2_, with the balance supplemented by 94% N_2_. The gas concentration was verified using an oxygen analyzer (Teledyne Electronic Technologies 60T) equipped with an oxygen sensor (OOM105, EnviteC-Wismar GmbH, Wismar, Germany).

### 4.5. Cell Viability Assay

A crystal violet assay was performed to determine the effect of drugs on cell viability under normoxic and hypoxic conditions. This colorimetric technique assesses adherent cell biomass, which directly correlates with the number of viable cells in adherent cultures. To perform this, 2 × 10^4^ cells were seeded per well in 48-well plates and incubated for 72 h under the culture conditions described in the “Cell Culture” section. The cells were treated with caffeine, metformin, and DCA concentrations specified in the “Drugs” section. At the end of the incubation period, the culture medium was aspirated from the wells to fix the cells with 1% glutaraldehyde for 20 min. Three washes with distilled water were then performed to remove excess glutaraldehyde. A 0.1% crystal violet solution was then added to each well and incubated for 20 min to allow the staining of the fixed cells. Excess dye was removed by thorough washing with distilled water, and the plates were allowed to dry. Once dry, the crystal violet incorporated into the cells was solubilized by adding 200 μL of 10% acetic acid to each well. The absorbance of each sample was measured at a wavelength of 540 nm using a Tecan Spectra Classic microplate reader (Männedorf, Switzerland). In total, 10% acetic acid was used as a blank, and a baseline control (with the plate at time zero) was included for data normalization. Eight wells were used per concentration of each drug, and four technical replicates were performed for each concentration. This resulted in 32 data points (n = 32) for the statistical analysis of each drug concentration derived from the four technical replicates of the eight wells.

### 4.6. Quantification of the Pharmacological Effect and Interaction Analysis

The pharmacological effect on cell viability was quantified by calculating the effect percentage, which was obtained from the percentage of cell viability normalized to the initial baseline absorbance. Specifically, the effect percentage is calculated as 100 minus the viability percentage (the absorbance of the treated well/the average baseline absorbance × 100). This method evaluated the magnitude of the cellular response to different drug concentrations, thus generating concentration–response curves to characterize the pharmacological effect.

In addition, isobolographic analysis was performed using the serial logarithmic concentrations of the drugs to determine the nature of their interaction (additive, synergistic, or antagonistic). This analysis identified concentration combinations that optimized (maximized or minimized) the effect on cell viability.

### 4.7. Statistical Analysis

Data with a normal distribution, as assessed by the Kolmogorov–Smirnov test, were expressed as the mean ± standard deviation (SD), while non-normal data were presented as the median and interquartile range (IQR). A *p*-value < 0.05 was considered statistically significant.

For the analysis of the concentration–response curves, given that the data did not follow a normal distribution, non-parametric tests were employed (Kruskal–Wallis for multiple comparisons, and the Mann–Whitney U test was employed for pairwise comparisons). In contrast, the isobologram data showed a normal distribution, and therefore, comparisons between the two groups were performed using Student’s *t*-test. To evaluate the effects of the same concentration under normoxia vs. hypoxia conditions, the Mann–Whitney U test was applied due to the absence of normality in these data. All analyses were performed using SPSS v26.0 software.

## 5. Conclusions

Clinically, these findings suggest the need to optimize combination therapies, develop personalized strategies based on the tumor microenvironment, and advance translational research to improve outcomes in cancer patients, especially those with tumor hypoxia. Metformin’s persistence of antitumor activity in hypoxia conditions makes it a valuable component in therapeutic strategies, and it could potentially improve the efficacy of treatments for tumors with this adverse condition.

## Figures and Tables

**Table 2:** The concentrations in mmol of the drug combinations metformin with caffeine and metformin with DCA were calculated to achieve a theoretical effect of 50% in HCC827 cells under normoxic and hypoxic conditions.

	Metformin	Caffeine	Metformin	Caffeine	Metformin	DCA	Metformin	DCA
1	7.59	2.13	5.59	3.69	7.59	2.02	5.58	4.61
2	3.79	1.06	3.79	1.84	3.79	1.01	2.79	2.30
3	1.89	0.53	1.89	0.92	1.89	0.50	1.39	1.15
4	0.94	0.26	0.94	0.46	0.94	0.25	0.69	0.57
	Normoxia		Hypoxia		Normoxia		Hypoxia	

## Data Availability

All data of interest are available for sharing, and you can access them by contacting the corresponding author.

## Supplementary Materials

The following supporting information can be downloaded at https://www.mdpi.com/article/10.3390/ijms26115014/s1.

## Abbreviations

The following abbreviations are used in this manuscript:

DCA	Dichloroacetate
mM	Millimolar
gr	Gram
U/mL	Units per milliliter
μg/mL	Micrograms per milliliter
mg/mL	Milligrams per milliliter
PBS	Phosphate-Buffered Saline
KCl	Potassium chloride
ATCC	American Type Culture Collection
DMEM	Dulbecco’s Modified Eagle Medium
EDTA	Ethylenediaminetetraacetic acid
μL	Microliter
mL	Milliliter
G	Grams
nM	Nanomolar
nm	Nanometer
IQR	Interquartile range
*p*-value	Probability value
HIF-1α	Hypoxia-inducible factor 1-alpha
mTOR	Mammalian target of rapamycin
Akt	Also known as protein kinase B (PKB)
c-MYC	Proto-oncogene c-Myc
VEGF	Vascular endothelial growth factor
MMP-2	Matrix metalloproteinase 2
AMPK	AMP-activated protein kinase
PI3K	Phosphatidylinositol-3 kinase
NSCLC	Non-small cell lung cancer
TKIs	Tyrosine Kinase Inhibitors
EGFR	Epidermal growth factor receptor
MDR-1	Multidrug resistance protein 1
NF-κB	Nuclear factor kappa B

## References

[1] Zhang Y., Vaccarella S., Morgan E., Li M., Etxeberria J., Chokunonga E., Manraj S.S., Kamate B., Omonisi A., Bray F. (2023). Global Variations in Lung Cancer Incidence by Histological Subtype in 2020: A Population-Based Study. Lancet Oncol..

[2] Leiter A., Veluswamy R.R., Wisnivesky J.P. (2023). The Global Burden of Lung Cancer: Current Status and Future Trends. Nat. Rev. Clin. Oncol..

[3] Zappa C., Mousa S.A. (2016). Non-Small Cell Lung Cancer: Current Treatment and Future Advances. Transl. Lung Cancer Res..

[4] Rodríguez-Lara V., Ramírez-Tirado L.A., Barrón F., Zatarain-Barrón Z.L., Flores-Estrada D., Arrieta O. (2019). Characteristics of Non-Small Cell Lung Cancer: Differences by Sex and Hormonal Status in a Mexican Population. Salud Publica Mex..

[5] Alatorre J.A., Campos-Gómez S., De la Mora E., Novick D., Cruz A., Iglesias-Chiesa J.M. (2020). Treatment Patterns and Costs Associated with Stage IV Non-Small Cell Lung Cancer in a Mexican Population: A Chart Review. Pharmacoecon. Open.

[6] Lu C.-C., Chiang J.-H., Tsai F.-J., Hsu Y.-M., Juan Y.-N., Yang J.-S., Chiu H.-Y. (2019). Metformin Triggers the Intrinsic Apoptotic Response in Human AGS Gastric Adenocarcinoma Cells by Activating AMPK and Suppressing mTOR/AKT Signaling. Int. J. Oncol..

[7] Lee J., Hong E.M., Kim J.H., Jung J.H., Park S.W., Koh D.H., Choi M.H., Jang H.J., Kae S.H. (2019). Metformin Induces Apoptosis and Inhibits Proliferation through the AMP-Activated Protein Kinase and Insulin-like Growth Factor 1 Receptor Pathways in the Bile Duct Cancer Cells. J. Cancer.

[8] Blandino G., Valerio M., Cioce M., Mori F., Casadei L., Pulito C., Sacconi A., Biagioni F., Cortese G., Galanti S. (2012). Metformin Elicits Anticancer Effects through the Sequential Modulation of DICER and C-MYC. Nat. Commun..

[9] Goetzman E.S., Prochownik E.V. (2018). The Role for Myc in Coordinating Glycolysis, Oxidative Phosphorylation, Glutaminolysis, and Fatty Acid Metabolism in Normal and Neoplastic Tissues. Front. Endocrinol..

[10] Mogavero A., Maiorana M.V., Zanutto S., Varinelli L., Bozzi F., Belfiore A., Volpi C.C., Gloghini A., Pierotti M.A., Gariboldi M. (2017). Metformin Transiently Inhibits Colorectal Cancer Cell Proliferation as a Result of Either AMPK Activation or Increased ROS Production. Sci. Rep..

[11] Zhou X., Chen J., Yi G., Deng M., Liu H., Liang M., Shi B., Fu X., Chen Y., Chen L. (2016). Metformin Suppresses Hypoxia-Induced Stabilization of HIF-1α through Reprogramming of Oxygen Metabolism in Hepatocellular Carcinoma. Oncotarget.

[12] Foukas L.C., Daniele N., Ktori C., Anderson K.E., Jensen J., Shepherd P.R. (2002). Direct Effects of Caffeine and Theophylline on P110 Delta and Other Phosphoinositide 3-Kinases. Differential Effects on Lipid Kinase and Protein Kinase Activities. J. Biol. Chem..

[13] Saiki S., Sasazawa Y., Imamichi Y., Kawajiri S., Fujimaki T., Tanida I., Kobayashi H., Sato F., Sato S., Ishikawa K.-I. (2011). Caffeine Induces Apoptosis by Enhancement of Autophagy via PI3K/Akt/mTOR/p70S6K Inhibition. Autophagy.

[14] He Z., Ma W.-Y., Hashimoto T., Bode A.M., Yang C.S., Dong Z. (2003). Induction of Apoptosis by Caffeine Is Mediated by the P53, Bax, and Caspase 3 Pathways. Cancer Res..

[15] Dong S., Kong J., Kong J., Shen Q., Kong F., Sun W., Zheng L. (2015). Low Concentration of Caffeine Inhibits the Progression of the Hepatocellular Carcinoma via Akt Signaling Pathway. Anticancer. Agents Med. Chem..

[16] Meisaprow P., Aksorn N., Vinayanuwattikun C., Chanvorachote P., Sukprasansap M. (2021). Caffeine Induces G0/G1 Cell Cycle Arrest and Inhibits Migration through Integrin Av, Β3, and FAK/Akt/c-Myc Signaling Pathway. Molecules.

[17] Allen K.T., Chin-Sinex H., DeLuca T., Pomerening J.R., Sherer J., Watkins J.B., Foley J., Jesseph J.M., Mendonca M.S. (2015). Dichloroacetate Alters Warburg Metabolism, Inhibits Cell Growth, and Increases the X-Ray Sensitivity of Human A549 and H1299 NSC Lung Cancer Cells. Free Radic. Biol. Med..

[18] Ancel J., Perotin J.-M., Dewolf M., Launois C., Mulette P., Nawrocki-Raby B., Dalstein V., Gilles C., Deslée G., Polette M. (2021). Hypoxia in Lung Cancer Management: A Translational Approach. Cancers.

[19] Kopecka J., Salaroglio I.C., Perez-Ruiz E., Sarmento-Ribeiro A.B., Saponara S., De Las Rivas J., Riganti C. (2021). Hypoxia as a Driver of Resistance to Immunotherapy. Drug Resist. Updat..

[20] Grabovsky Y., Tallarida R.J. (2004). Isobolographic Analysis for Combinations of a Full and Partial Agonist: Curved Isoboles. J. Pharmacol. Exp. Ther..

[21] Huang R.-Y., Pei L., Liu Q., Chen S., Dou H., Shu G., Yuan Z.-X., Lin J., Peng G., Zhang W. (2019). Isobologram Analysis: A Comprehensive Review of Methodology and Current Research. Front. Pharmacol..

[22] Ashinuma H., Takiguchi Y., Kitazono S., Kitazono-Saitoh M., Kitamura A., Chiba T., Tada Y., Kurosu K., Sakaida E., Sekine I. (2012). Antiproliferative Action of Metformin in Human Lung Cancer Cell Lines. Oncol. Rep..

[23] Ko J.-C., Huang Y.-C., Chen H.-J., Tseng S.-C., Chiu H.-C., Wo T.-Y., Huang Y.-J., Weng S.-H., Chiou R.Y.Y., Lin Y.-W. (2013). Metformin Induces Cytotoxicity by Down-Regulating Thymidine Phosphorylase and Excision Repair Cross-Complementation 1 Expression in Non-Small Cell Lung Cancer Cells. Basic. Clin. Pharmacol. Toxicol..

[24] Ma Y., Guo F.-C., Wang W., Shi H.-S., Li D., Wang Y.-S. (2013). K-ras Gene Mutation as a Predictor of Cancer Cell Responsiveness to Metformin. Mol. Med. Rep..

[25] Xie W., Wang L., Sheng H., Qiu J., Zhang D., Zhang L., Yang F., Tang D., Zhang K. (2017). Metformin Induces Growth Inhibition and Cell Cycle Arrest by Upregulating MicroRNA34a in Renal Cancer Cells. Med. Sci. Monit..

[26] Lin H., Zhou W., Huang Y., Ren M., Xu F., Wang H. (2020). Systemic Hypoxia Potentiates Anti-Tumor Effects of Metformin in Hepatocellular Carcinoma in Mice. Acta Biochim. Biophys. Sin..

[27] Gude R.P., Menon L.G., Rao S.G. (2001). Effect of Caffeine, a Xanthine Derivative, in the Inhibition of Experimental Lung Metastasis Induced by B16F10 Melanoma Cells. J. Exp. Clin. Cancer Res..

[28] Merighi S., Benini A., Mirandola P., Gessi S., Varani K., Simioni C., Leung E., Maclennan S., Baraldi P.G., Borea P.A. (2007). Caffeine Inhibits Adenosine-Induced Accumulation of Hypoxia-Inducible Factor-1alpha, Vascular Endothelial Growth Factor, and Interleukin-8 Expression in Hypoxic Human Colon Cancer Cells. Mol. Pharmacol..

[29] Eini H., Frishman V., Yulzari R., Kachko L., Lewis E.C., Chaimovitz C., Douvdevani A. (2015). Caffeine Promotes Anti-Tumor Immune Response during Tumor Initiation: Involvement of the Adenosine A2A Receptor. Biochem. Pharmacol..

[30] Wang G., Bhoopalan V., Wang D., Wang L., Xu X. (2015). The Effect of Caffeine on Cisplatin-Induced Apoptosis of Lung Cancer Cells. Exp. Hematol. Oncol..

[31] Lu X., Zhou D., Hou B., Liu Q.-X., Chen Q., Deng X.-F., Yu Z.-B., Dai J.-G., Zheng H. (2018). Dichloroacetate Enhances the Antitumor Efficacy of Chemotherapeutic Agents via Inhibiting Autophagy in Non-Small-Cell Lung Cancer. Cancer Manag. Res..

[32] Al-Azawi A., Sulaiman S., Arafat K., Yasin J., Nemmar A., Attoub S. (2021). Impact of Sodium Dichloroacetate Alone and in Combination Therapies on Lung Tumor Growth and Metastasis. Int. J. Mol. Sci..

[33] Li B., Li X., Ni Z., Zhang Y., Zeng Y., Yan X., Huang Y., He J., Lyu X., Wu Y. (2016). Dichloroacetate and Metformin Synergistically Suppress the Growth of Ovarian Cancer Cells. Oncotarget.

[34] Kolesnik D.L., Pyaskovskaya O.N., Boichuk I.V., Solyanik G.I. (2014). Hypoxia Enhances Antitumor Activity of Dichloroacetate. Exp. Oncol..

[35] Sanchez W.Y., McGee S.L., Connor T., Mottram B., Wilkinson A., Whitehead J.P., Vuckovic S., Catley L. (2013). Dichloroacetate Inhibits Aerobic Glycolysis in Multiple Myeloma Cells and Increases Sensitivity to Bortezomib. Br. J. Cancer.

[36] Popović D.J., Lalošević D., Miljković D., Popović K.J., Čapo I., Popović J.K. (2018). Caffeine Induces Metformin Anticancer Effect on Fibrosarcoma in Hamsters. Eur. Rev. Med. Pharmacol. Sci..

[37] Kim T.S., Lee M., Park M., Kim S.Y., Shim M.S., Lee C.Y., Choi D.H., Cho Y. (2021). Metformin and Dichloroacetate Suppress Proliferation of Liver Cancer Cells by Inhibiting mTOR Complex 1. Int. J. Mol. Sci..

[38] Hong S.-E., Jin H.-O., Kim H.-A., Seong M.-K., Kim E.-K., Ye S.-K., Choe T.-B., Lee J.K., Kim J.-I., Park I.-C. (2016). Targeting HIF-1α Is a Prerequisite for Cell Sensitivity to Dichloroacetate (DCA) and Metformin. Biochem. Biophys. Res. Commun..

[39] Yin X., Wei Z., Song C., Tang C., Xu W., Wang Y., Xie J., Lin Z., Han W. (2018). Metformin Sensitizes Hypoxia-Induced Gefitinib Treatment Resistance of HNSCC via Cell Cycle Regulation and EMT Reversal. Cancer Manag. Res..

[40] Wang J.-C., Li G.-Y., Li P.-P., Sun X., Li W.-M., Li Y., Lu S.-Y., Liu P.-J. (2017). Suppression of Hypoxia-Induced Excessive Angiogenesis by Metformin via Elevating Tumor Blood Perfusion. Oncotarget.

[41] Weidemann A., Johnson R.S. (2008). Biology of HIF-1alpha. Cell Death Differ..

[42] Cornelissen J.J., Sonneveld P., Schoester M., Raaijmakers H.G., Nieuwenhuis H.K., Dekker A.W., Lokhorst H.M. (1994). MDR-1 Expression and Response to Vincristine, Doxorubicin, and Dexamethasone Chemotherapy in Multiple Myeloma Refractory to Alkylating Agents. J. Clin. Oncol..

[43] Chen G.K., Durán G.E., Mangili A., Beketic-Oreskovic L., Sikic B.I. (2000). MDR 1 Activation Is the Predominant Resistance Mechanism Selected by Vinblastine in MES-SA Cells. Br. J. Cancer.

[44] Friche E., Skovsgaard T., Nissen N.I. (1989). Anthracycline Resistance. Acta Oncol..

[45] Asakuno K., Kohno K., Uchiumi T., Kubo T., Sato S., Isono M., Kuwano M. (1994). Involvement of a DNA Binding Protein, MDR-NF1/YB-1, in Human MDR1 Gene Expression by Actinomycin D. Biochem. Biophys. Res. Commun..

[46] Yusuf R.Z., Duan Z., Lamendola D.E., Penson R.T., Seiden M.V. (2003). Paclitaxel Resistance: Molecular Mechanisms and Pharmacologic Manipulation. Curr. Cancer Drug Targets.

[47] Swietach P., Vaughan-Jones R.D., Harris A.L., Hulikova A. (2014). The Chemistry, Physiology and Pathology of pH in Cancer. Philos. Trans. R. Soc. Lond. B Biol. Sci..

[48] Thews O., Riemann A., Nowak M., Gekle M. (2014). Impact of Hypoxia-Related Tumor Acidosis on Cytotoxicity of Different Chemotherapeutic Drugs in Vitro and in Vivo. Adv. Exp. Med. Biol..

[49] Cheng Z.-X., Wang D.-W., Liu T., Liu W.-X., Xia W.-B., Xu J., Zhang Y.-H., Qu Y.-K., Guo L.-Q., Ding L. (2014). Effects of the HIF-1α and NF-κB Loop on Epithelial-Mesenchymal Transition and Chemoresistance Induced by Hypoxia in Pancreatic Cancer Cells. Oncol. Rep..

[50] Chauhan S.S., Toth R.K., Jensen C.C., Casillas A.L., Kashatus D.F., Warfel N.A. (2020). PIM Kinases Alter Mitochondrial Dynamics and Chemosensitivity in Lung Cancer. Oncogene.

[51] Marayati R., Stafman L.L., Williams A.P., Bownes L.V., Quinn C.H., Aye J.M., Stewart J.E., Yoon K.J., Anderson J.C., Willey C.D. (2021). PIM Kinases Mediate Resistance to Cisplatin Chemotherapy in Hepatoblastoma. Sci. Rep..

[52] Zhang J., Chen G., Gao Y., Liang H. (2020). HOTAIR/miR-125 Axis-Mediated Hexokinase 2 Expression Promotes Chemoresistance in Human Glioblastoma. J. Cell Mol. Med..

[53] Kuznetsov A.V., Veksler V., Gellerich F.N., Saks V., Margreiter R., Kunz W.S. (2008). Analysis of Mitochondrial Function in Situ in Permeabilized Muscle Fibers, Tissues and Cells. Nat. Protoc..

