# Supplementary material for: Modulation of the Antitumor Response to Metformin, Caffeine, and Sodium Dichloroacetate by the Hypoxic Microenvironment in Lung Cancer Cells
Source: Int J Mol Sci. 2025 May 23;26(11):5014. doi: 10.3390/ijms26115014 (PMC12155099; doi:10.3390/ijms26115014)
Supplement: Supplementary file 1 [file ijms-26-05014-s001.zip › ijms-3611914-supplementary.pdf]

Annex 1

Table S1. Metformin under Normoxia (Wilcoxon test):

Concentration 1 (mM/L)	Concentration 2 (mM/L)	p-value
1.8	3.2	0.262618
1.8	5.6	0.109745
1.8	10	0.007646
1.8	18	0.01796
1.8	32	0.003346
3.2	5.6	0.161429
3.2	10	0.075368
3.2	18	0.027992
3.2	32	0.003346
5.6	10	0.440867
5.6	18	0.027992
5.6	32	0.007686
10	18	0.017756
10	32	0.002218
18	32	0.799495

Table S2. Metformin under Hypoxia (Wilcoxon test):

Concentration 1 (mM/L)	Concentration 2 (mM/L)	p-value
1.8	3.2	0.878482
1.8	5.6	0.016605
1.8	10.0	0.020795
1.8	18.0	0.012515
1.8	32.0	0.002218
3.2	5.6	0.028402
3.2	10.0	0.114128
3.2	18.0	0.015156
3.2	32.0	0.005062
5.6	10.0	0.858955
5.6	18.0	0.020879
5.6	32.0	0.005062
10.0	18.0	0.016605
10.0	32.0	0.003346
18.0	32.0	0.004439

Table S3: Inter-group analysis in metformin (environmental condition: normoxia vs hypoxia) using the Mann-Whitney U test.

Concentration (mM/L) Normoxia	Concentration (mM/L) Hypoxia	p-value
1.8	1.8	0.622
3.2	1.8	0.398
5.6	1.8	0.87
10	1.8	0.355
18	1.8	0.077
31	1.8	0.772

Table S4. Caffeine under Normoxia (Wilcoxon test):

Concentration 1 (mM/L)	Concentration 2 (mM/L)	p-value
0.5	1.0	0.130665
0.5	1.8	0.139414
0.5	3.2	0.004276
0.5	5.6	0.000982
0.5	10.0	0.000982
1.0	1.8	0.286003
1.0	3.2	0.004742
1.0	5.6	0.002218
1.0	10.0	0.002218
1.8	3.2	0.002218
1.8	5.6	0.002218
1.8	10.0	0.002218
3.2	5.6	0.000196
3.2	10.0	0.002293
5.6	10.0	0.081470

Table S5. Caffeine under Hypoxia (Wilcoxon test):

**Concentration 1 (mM/L)**	**Concentration 2 (mM/L)**	**p-value**
0.5	1	0.111
0.5	1.8	0.123
0.5	3.2	0.148
0.5	5.6	0.002
0.5	10	0.01
1	1.8	0.463
1	3.2	0.011
1	5.6	0.001
1	10	0.004
1.8	3.2	0.004
1.8	5.6	0.000421
1.8	10	0.000982
3.2	5.6	0.003599
3.2	10	0.000655
5.6	10	0.172

Table S6: Inter-group analysis in Caffeine (environmental condition: normoxia vs hypoxia) using the Mann-Whitney U test.

**Concentration 1 (mM/L) Normoxia**	**Concentration 2 (mM/L) Hypoxia**	**p-value**
0.5	0.5	1
1	0.5	0.397
1.8	0.5	0.964
3.2	0.5	0.008
5.6	0.5	2.96E-07
10	0.5	0.000001

Table S7: DCA under Normoxia (Wilcoxon test):

Concentration 1 (mM/L)	Concentration 2 (mM/L)	p-value
1	5.6	0.423
1	10	0.952
1	40	0.016
1	56.2	0.003
1	100	0.003
5.6	10	0.441
5.6	40	0.003
5.6	56.2	0.002
5.6	100	0.002
10	40	0.007
10	56.2	0.007
10	100	0.007
40	56.2	0.346
40	100	0.002
56.2	100	0.002

Table S8: DCA under Hypoxia (Wilcoxon test):

Concentration 1 (mM/L)	Concentration 2 (mM/L)	p-value
1	5.6	0.767
1	10	0.656
1	40	0.017
1	56.2	0.018
1	100	0.003
5.6	10	0.767
5.6	40	0.043
5.6	56.2	0.007
5.6	100	0.007
10	40	0.017
10	56.2	0.004
10	100	0.003
40	56.2	0.498
40	100	0.027
56.2	100	0.05

Table S9: Inter-group analysis in DCA (environmental condition: normoxia vs hypoxia) using the Mann-Whitney U test.

Concentration (mM/L) Normoxia	Concentration (mM/L) Hypoxia	p-value
1.0	1.0	0.218
1.0	5.6	0.046
1.0	10.0	0.239
1.0	40.0	0.554
1.0	56.2	0.043
1.0	100.0	0.036

Table S10: ISOBOLOGRAM METFORMIN + CAFFEINE in NORMOXIA

	Value	S.E.M (+/-)	CI
Interaction index (γ)=	**0.90**	0.34	0.39 - 2.12
EC50 Theoretical=	7.85	0.63	6.29 - 9.81
EC50 Experimental=	7.09	2.65	1.42 - 35.42
t Student=	***p>0.05 Additivity***		

Table S11: ISOBOLOGRAM METFORMIN + CAFFEINE in HYPOXIA

	Value	S.E.M (+/-)	CI
Interaction index (γ)=	**2.88**	0.90	1.43 - 5.80
EC50 Theoretical=	9.28	0.69	7.53 - 11.43
EC50 Experimental=	26.72	7.15	7.18 - 99.42
t Student=	***p<0.05 Antagonism***		

Table S12: ISOBOLOGRAM METFORMIN + DCA in NORMOXIA

	Value	S.E.M (+/-)	CI
Interaction index (γ)=	**0.98**	0.35	0.44 - 2.17
EC50 Theoretical=	7.70	0.59	6.21 - 9.56
EC50 Experimental=	7.55	2.62	1.69 - 33.71
t Student=	***p>0.05 Additivity***		

Table S13: ISOBOLOGRAM METFORMIN + DCA in HYPOXIA

	Value	S.E.M (+/-)	CI
Interaction index (γ)=	**4.20**	1.44	1.95 - 9.05
EC50 Theoretical=	10.21	0.77	8.27 - 12.60
EC50 Experimental=	42.92	14.38	10.13 - 81.85
t Student=	***p<0.05 Antagonism***		
